# Determining Causation from Observational Studies: A Challenge for Modern Neuroepidemiology

**DOI:** 10.3389/fneur.2017.00265

**Published:** 2017-06-01

**Authors:** George A. Jelinek

**Affiliations:** ^1^University of Melbourne, Melbourne, VIC, Australia

**Keywords:** Mendelian randomization analysis, neuroepidemiology, specialty grand challenge, multiple sclerosis, randomized control trials, vitamin D, lifestyle risk factors

## Background

While epidemiology is the study of frequencies, trends, and determinants of disease in specified populations, the overriding aim of epidemiology is to apply such knowledge to prevention. Both primary and secondary prevention benefit from a detailed understanding of risk factors that can be uncovered through careful epidemiological research. Clearly some factors associated with outcomes in cross-sectional studies may be risk factors, but also may not, and indeed may not be in longitudinal studies. While risk factors are causal, some are modifiable and some not. But some risk factors that we have long regarded as fixed and not modifiable, such as genetics, have more recently, in the exploding science of epigenetics, been shown to be more or less expressed through different lifestyles. Ornish et al. has shown for example that intensive lifestyle changes favorably modulate gene expression in prostate cancer (1) and, with Nobel laureate Elizabeth Blackburn, that such environmental factors can reverse what was thought to be an inevitable decline in telomere length associated with aging and disease (2).

## Newer Techniques in Neuroepidemiology

Comprehensive analysis of modifiable risk factors through epidemiological studies paves the way for translation into appropriate intervention studies. But such studies are difficult. However, new techniques in observational epidemiology are now transforming our capacity to infer causality and may well offer some advantages over conventional randomized controlled trials (RCTs). New analytic approaches enable us to better account for biases including selection, confounding, and information bias. While longitudinal cohort studies have traditionally allowed stronger inferences than cross-sectional studies, more recent techniques such as instrumental variable analysis, which better accounts for confounding and reverse causation, offer great potential to improve causal inference. Key assumptions of instrumental variable analysis are that the instrument is only related to the outcome through the exposure (or risk factor) and that there are no direct paths between the instrument and confounders. Genetic polymorphisms are excellent examples of instrumental variables in epidemiological studies since they are “randomly assigned” and have been used in a range of studies of lifestyle risk factors such as obesity. The use of genetic variants as instruments is referred to as Mendelian randomization (3).

## The Example of Multiple Sclerosis (MS)

Let us take MS as an example. MS is thought to be an autoimmune inflammatory demyelinating disease of the central nervous system. The genetic predisposition to MS accounts for around 24% of the risk of developing the disease (4), and the likely environmental risk factors have been well known for many years, elucidated through epidemiological research. Those factors about which there is a reasonable degree of agreement include low vitamin D levels (5) and lack of sunlight exposure (6), cigarette smoking (7), low omega 3 fatty acid intake (8) and poor blood lipid profile (9), lack of exercise (10), and obesity (11). While findings have generally been consistent with respect to these risk factors, many studies have not adequately accounted for confounding or reverse causation.

While some or all of these factors are likely to be causally related to disease development, for many years, it was thought that there was little environmental influence on progression. However, recent genome-wide studies in people with MS have shown that genetics plays little role in disease progression to disability (12). This has prompted renewed search for modifiable lifestyle risk factors that may accelerate disability progression with the aim of allowing a comprehensive secondary prevention program to be developed for people with the illness. It is biologically plausible that some or most of the environmental risk factors for progression would be the same as those that precipitate the disease in the first place; studies conducted over many decades now have pointed the finger at animal fats and poor fruit and vegetable intake in the diet (13, 14), poor blood lipid profile (15), low omega 3 fatty acid intake (13, 16), obesity (9), low vitamin D levels (17), lack of sun exposure (18), cigarette smoking (17, 19), low levels of exercise (20), and stress (21).

## Observational Versus Intervention Data

The problem for researchers is how and indeed whether it is feasible to proceed beyond observational to intervention data, given that the majority of interventions based on these data require significant lifestyle change by research participants. It is at least arguable that more sophisticated epidemiological techniques may make this requirement moot. The example of Mendelian randomization in proving the effect of low blood vitamin D levels in causing MS is illustrative. Richards’ group from McGill University’s Department of Epidemiology elegantly applied genome-wide data on genetic variants that predicted blood vitamin D levels from the Canadian Multicentre Osteoporosis Study to participants in the International MS Genetics Consortium study (22). They found that a genetic decrease in blood vitamin D level predicted increased MS susceptibility, effectively meaning that a 50% increase in blood level decreased the odds of getting MS by 50%.

This sophisticated research largely removed the possibility of confounding or reverse causation. While some may undervalue this evidence because it is observational, it actually has important advantages over RCTs, long considered the research gold standard. Mendelian randomization techniques allow for the lifetime exposure to vitamin D-lowering genes in the population, whereas RCTs are by necessity shorter and generally on smaller populations. Richards’ study provides strong evidence of causation, as did their later study on genetically determined obesity and MS risk (11), and backs up prospective observational studies such as the US Nurses Health Study that showed significantly reduced risk of developing MS with relatively low doses of vitamin D supplementation (23). What is needed is a similar focus, through similar methodology, on the association, for example, of this genetically determined propensity to obesity with disease progression. Arguably, such epidemiological studies could provide more robust evidence than intervention studies, given their limitations. This is possible for a range of neurological diseases with significant lifestyle determinants.

## Problems with RCTs

Randomized controlled trials of disease-modifying drugs (DMDs) in MS are relatively simple to perform and have contributed to major therapeutic advances in MS management. While there are some limitations around blinding related to side effects, randomization is relatively simple and adherence is not the difficult issue for DMD research that it is in lifestyle risk factor modification studies. Take diet for example, which is probably one of the most studied but most controversial of risk factors for MS progression. Since the uncontrolled intervention study of Swank and Dugan over an extraordinarily long timeframe showed that “poor dieters” had much worse outcomes than those who could dramatically reduce animal fat in their diets (24), numerous epidemiological studies (25) and a few very small RCTs (26, 27) have provided evidence about animal fat being a key risk in MS disease progression, along with resultant poor blood lipid profile (15, 28, 29) and overweight and obesity (11, 30).

However, RCTs that test these interventions are difficult to conduct and have significant limitations (31). First, loss to follow-up is an important issue, particularly loss of participants with more severe disease or more rapid disease progression. This tends to exaggerate treatment effects in those who remain to study conclusion. Second, lack of adherence to the study treatment is an obvious limitation in determining causal effects of treatment. While an “as treated” analysis can be useful, it introduces bias, and intention-to-treat (“as allocated”) analysis is usually preferred, despite its inherent underestimation of causal treatment effect. Finally, unblinding, which is unavoidable in complex interventions where the goal is behavior change, encourages behavior change outside of the prescribed intervention, with unpredictable effects of treatment effect estimation depending on what behaviors change and in which group.

## A Challenge for Neuroepidemiology

Given these and other limitations of RCTs in this area, the challenge for modern neuroepidemiology is to further develop techniques that allow strong causal inferences to be drawn from more sophisticated longitudinal observational research to allow the framing of robust secondary preventive recommendations for people with potentially devastating neurological illnesses. The risks of waiting for RCTs to “prove” the case for risk factor modification-based secondary prevention are obvious.

## Author Contributions

The author confirms being the sole contributor of this work and approved it for publication.

## Conflict of Interest Statement

The author receives royalties from his book Overcoming Multiple Sclerosis: The Evidence-Based 7 Step Recovery Program.
